# Assessing and Improving the Quality in Mental Health Services

**DOI:** 10.3390/ijerph17010249

**Published:** 2019-12-30

**Authors:** Lampros Samartzis, Michael A. Talias

**Affiliations:** 1 Faculty of Economics and Management, Open University of Cyprus, Latsia, Nicosia, Cyprus; 2 Department of Psychiatry, Medical School, University of Cyprus, Nicosia, Cyprus; 3 Mental Health Services, Athalassa Psychiatric Hospital, Nicosia, Cyprus

**Keywords:** mental health and quality, mental health services and effectiveness, psychiatric treatment, psychiatry, medicine, psychology, efficiency, sustainability

## Abstract

Background: The mental health of the population consists of the three essential pillars of quality of life, economy, and society. Mental health services take care of the prevention and treatment of mental disorders and through them maintain, improve, and restore the mental health of the population. The purpose of this study is to describe the methodology for qualitative and quantitative evaluation and improvement of the mental health service system. Methods: This is a narrative review study that searches the literature to provide criteria, indicators, and methodology for evaluating and improving the quality of mental health services and the related qualitative and quantitative indicators. The bibliography was searched in popular databases PubMed, Google Scholar, CINAHL, using the keywords “mental”, “health”, “quality”, “indicators”, alone or in combinations thereof. Results: Important quality indicators of mental health services have been collected and presented, and modified where appropriate. The definition of each indicator is presented here, alongside its method of calculation and importance. Each indicator belongs to one of the eight dimensions of quality assessment: (1) Suitability of services, (2) Accessibility of patients to services, (3) Acceptance of services by patients, (4) Ability of healthcare professionals to provide services, (5) Efficiency of health professionals and providers, (6) Continuity of service over time (ensuring therapeutic continuity), (7) Efficiency of health professionals and services, (8) Safety (for patients and for health professionals). Discussion/Conclusions: Accessibility and acceptability of service indicators are important for the attractiveness of services related to their use by the population. Profitability indicators are important economic indicators that affect the viability and sustainability of services, factors that are now taken into account in any health policy. All of the indicators mentioned are related to public health, affecting the quality of life, morbidity, mortality, and life expectancy, directly or indirectly. The systematic measurement and monitoring of indicators and the measurement and quantification of quality through them, are the basis for evidence-based health policy for improvement of the quality of mental health services.

## 1. Introduction

The burden of illnesses from mental health disorders is by far the highest of all health problems worldwide, accounting for 13% of the total burden of illness from all diseases. More specifically, mental illness accounts for 32.4% of years lost due to mental illness or disability (YLDs) and 13% of disability-adjusted life years (DALYs), which is the exact measure of disease burden [1]. DALYs corresponding to the burden of mental illness is the sum of YLDs along with years lost due to premature death from mental illness (YLL). At the EU Member State level, the cost of mental disorders is estimated at 3–4% of GDP, mainly due to the loss of productivity [2]. Providing high-quality psychiatric care to mental patients, especially in the context of the EU, is an obligation of the welfare state, a duty of the mental health professionals, and the patients’ right. 

The unmet need for quality in mental health services that responds to patients’ needs and respect citizens’ right to mental health can be achieved over time through strategic planning, annual evaluation and targeted improvement of quality in the provided mental health services. In addition, a qualitative and quantitative evaluation with indicators of quality in mental health services is needed, in order to identify and reveal the needs of disadvantaged social groups, to facilitate the state intervening politically to ensure universal and equal access to services for the population [3].

Evaluation/measurement of existing quality is a prerequisite for its improvement: What cannot be evaluated and measured cannot be improved. The evaluation of the quality of mental health services at the national and international levels is qualitative and quantitative. However, this is a complicated process as there are no common scientific language, objectives, and priorities and there are no common examples in this area, and for most countries—even for those that are developed—there are clearly unmet needs in this area [4].

There are significant differences in the quality of mental health services both between and within countries, which clinically take the form of different distances, within each health system, between the clinical guidelines of international scientific societies and daily clinical practice. Clinical decisions about the same symptom and illness differ from country to country because the systems in place for psychiatry are different, and therefore the resources and opportunities for quality mental health care are different.

Studies evaluating the quality of interventions show that, as a rule, daily clinical practice is always below the level set by national and international guidelines [5]. The differences in adherence to the guidelines are therefore explained as well as the quality indicators. Focusing on evaluation and quality improvement can reduce the heterogeneity of clinical decisions and optimize the outcome of cases treated, to some extent, without overlooking the fact that that clinical medicine is not only a science but also an art, as described in the Hippocratic Oath.

For quality assessment in health systems, there are some useful weighted generic tools that may initially help [6], such as the WHO Assessment Instrument for Mental Health Systems (WHO-AIMS) [7] and WHO-Quality Rights [8], although these are not available either in Greek or in many other languages. Their contribution consists of a general descriptive assessment of the quality of the mental health sector of health systems.

In this study, we propose and describe the methodology and procedure for a thorough evaluation of the quality of mental health services at the health system level. Evaluation is based on evaluation criteria, and each criterion has its own specific evaluation indicators. Different criteria mean different problems. The indicators do not suggest a solution to the problem under consideration. Indicators collectively can help to clarify and quantify policy objectives and strategies for an optimized Mental Health System.

The purpose of the project is twofold: first, to propose an integrated model of mental health services evaluation, and second to target specific interventions to improve the quality of mental health services at the level of the health system.

## 2. Materials and Methods

In order to search for criteria, indicators and methodology for evaluating and improving the quality of mental health services and related qualitative and quantitative indicators, a bibliography was searched in large databases PubMed, Google Scholar, CINAHL, using the keywords “mental”, “health”, “quality”, “indicators”, alone or in combinations thereof. Peer review journals were searched with English keywords for articles published in peer-reviewed journals possessing an English abstract without setting restrictions for the languages of the main text. Mental health databases maintained by Eurostat, WHO, and OECD were also searched.

To evaluate and measure the quality of mental health services, we propose and describe an eight-dimensional model in this study. Each dimension is an independent, quality assessment criterion. The model comes from the Canadian Health System General Rating System but is modified and enriched by the author specifically for mental health services and includes only mental health-related indicators [9]. Other authors have used simplified three-dimensional models [10] where all dimensions are concentrated in three categories: (1) clinical improvement, (2) patient safety, (3) overall patient experience of treatment/care, which qualifies for evaluation in structural/clinical level, but is not sufficiently detailed to assess at a macroscopic level the mental health system. We consider that the quality evaluation should be multidimensional and interdisciplinary. Therefore, it was considered necessary to include financial dimensions as well, since any development of a new service is also assessed at a cost, sustainability, and sustainable development level.

The eight dimensions (criteria) in which mental health services are evaluated are as follows: (1) appropriateness of services provided, (2) accessibility of patients to services provided, (3) acceptability of the services from the patients, (4) competence of mental health care providers, (5) effectiveness of mental health professionals (6) therapeutic continuity in the mental health system, (7) the efficiency of health professionals, (8) the safety of patients and health care providers. Indicators of each dimension can be characterized as indicators of structure, indicators of process, and indicators of the outcome, as suggested by other researchers in the evaluation of the quality of mental health services [6].

Each of the dimensions has aspects called indicators, which are described in detail in the text. Specifically, for each indicator is described (a) the background and the general meaning of the index, (b) the definition that includes how the index is calculated, and (c) the performance target for mental health services, quantitatively or qualitatively: The performance target is the benchmark. 

## 3. Results

### 3.1. Appropriateness Criterion, or Appropriateness Dimension. Indicators of Appropriateness of Mental Health Services

#### 3.1.1. The Number of Chronic Patients Hospitalized in Psychiatric Hospitals Rather Than Rehabilitation in an Outpatient Setting

This is a structural indicator of the mental health system, which indicates the adequacy or inadequacy of outpatient psychiatric structures created in parallel with deinstitutionalization. During the long period of deinstitutionalization, most patients with chronic mental disorders were discharged from psychiatric hospitals who had been staying for months or years and referred for psychiatric care to community mental health units as outpatients. The definition of the chronic disease, as well as the relevant references, has now been added in the manuscript. Chronic disease is one lasting three months or more, by the definition of the U.S. National Center for Health Statistics [11,12]. A patient has to be hospitalized for at least three months in order to be considered for chronic hospitalization.

These patients at best are now living in independent or sheltered apartments, alone, with friends or relatives, and in many cases they reside in approved hostels and are monitored by a social worker, community nurse, and community nursing psychiatrist. Psychiatric hospitals shrank in the number of beds and have changed shape and use: They have maintained short-stay clinics, day-care units, emergency clinics, and rehabilitation clinics, while closing long-term clinics that were replaced by less isolated community services, not only for patients with mental disorders but also for patients with developmental disabilities.

This indicator is calculated as a fraction where the numerator is the number of patients with chronic mental disorders and those with developmental disabilities living in the psychiatric hospital, and the denominator is the total number of patients in the hospital.

The performance target is to reduce gradually, year by year, patients who are permanently hospitalized while being transferred to community services. Therefore, this indicator can be used to evaluate the progress of a hospital year on year, as long as the indicator is calculated on an annual basis. The indicator can also be used for comparison between countries with similar psychiatric therapeutic cultures and deinstitutionalization approach, as is the case in the EU Member States. Note that the purpose of this indicator is to evaluate mental health services with the aim of improving them and thereby improving the quality of life of patients. For this to happen, it is not enough to dismiss patients from the psychiatric clinic, but also to provide adequate community mental health structures. The indicator should, therefore, be assessed against the adequacy of community structures, as otherwise, the indicator may improve (with the closure of psychiatric clinics and the dismissal of patients), but the quality of life, morbidity, and mortality of some patients may deteriorate if there is not sufficient development of community structures.

#### 3.1.2. Number of Cases That Could Avoid Admission to Hospital with Appropriate External Intervention

This is a structural indicator, focusing on assessing the adequacy of appropriate outpatient mental health structures. In the mental health system, there are a number of patients admitted to the hospital (one or more times) per year due to the lack of availability of corresponding outpatient services. Unnecessary hospital admission and hospitalization are costly to the system and not necessarily beneficial to the patient as the patient does not receive proper care.

This indicator is a fraction where the numerator is the number of inpatients admitted due to inadequate outpatient structure and intervention, while the denominator is the number of inpatients patients treated in the same clinic during the same time period.

Obviously, the performance target is that the fraction becomes smaller and smaller with each passing year. The larger the fraction, the greater the inadequacy of outpatient structures (e.g., community psychiatry, community nursing). At the system level, the indicator can be used to compare the adequacy of outpatient mental health care between the EU Member States.

#### 3.1.3. Number of Cases Treated without Indication

This is an indicator that assesses the structure of Mental Health Services and is indicative of the availability of specific structures. In particular, it assesses whether the patient is treated in the appropriate setting (hospitalized or outpatient), that is, if he or she receives appropriate personalized treatment. In psychiatry, it is very important that the treatment provided is tailored to the individual bio-psychosocial needs of each patient. Therefore, the psychosocial context is always taken into account. Inappropriate treatment may be due to the lack of this structure (e.g., lack of a section on eating disorders) or the completeness of this structure (e.g., patient being admitted to a psychiatric hospital due to the fullness of the psychiatric ward of the general hospital) or due to lack of specialization and unclear roles between departments (e.g., a patient with depression and alcohol dependence may be hospitalized (1) in a special alcohol dependence and rehabilitation clinic, or (2) a general hospital’s psychiatric clinic, or (3) in the psychiatric hospital, or (4) in internal medicine ward of the general hospital).

This indicator is calculated as a fraction where the number is the number of patients who received appropriate care in the clinic who were hospitalized over a period of one year. The denominator is the total number of patients treated in the same clinic during the same period.

The performance objective is that all patients receive appropriate treatment, that is, the fraction value as close to the unit as possible. The indicator should be calculated on a yearly basis to show the course of improvement of the health system over time, for example, by comparing this year’s score with the last year’s score. It can also be used at the level of an individual clinic, which shows the extent to which the clinic is treating cases that are not of its specialty (e.g., non-psychotic patients that are voluntarily hospitalized in the psychiatric hospital due to lack of beds in the psychiatric clinic of the general hospital or psychiatric clinic). The indicator can also be used to compare health systems between them, e.g., between the EU Member States.

### 3.2. Accessibility Criterion, or Otherwise Accessibility Dimensions. Accessibility and Accessibility Indicators

#### 3.2.1. Waiting Time at the Accident and Emergency Department

It is important for prognostic reasons that the mentally ill patient who comes to the Accident and Emergency Department (A&E) does not have to wait long until he or she is examined by the A&E physician and later by the psychologist. Long waiting may be associated with a worsening of the prognosis in some cases. Some patients who come to A&E may be at risk due to acute medical disease or worsening chronic disease. Especially for those suffering from an acute psychiatric disorder, long waiting times can lead to a particularly adverse development, especially for patients with aggression, self-harm, or suicidality.

This is a process indicator. The indicator is a fraction where the numerator is the number of patients who came to the A&E and served in less than 4 h, and the denominator is the total number of patients admitted to the A&E over the same period of one year.

The performance target published by the National Health System can be used as a reference. Specifically, the UK’s National Health System (NHS) has set a target of 95% of patients admitted in A&E to be served there in less than 4 h [13]. This does not just mean starting the examination, but that within four hours the patient has to be fully managed by the A&E, that is, the patient has been admitted, or referred to another unit or discharged by the Accident and Emergency Department to go home. However, even the NHS itself cannot fully meet this self-imposed target, as only 75% of cases are fully managed within 4 h [14].

#### 3.2.2. Waiting Days for Evaluation in the Outpatient Clinic

This is a process indicator. Citizens have equal rights to health, and this means that the health system must provide citizens equal access and coverage. There are many factors that may reduce citizens’ access to the health system, but the most common and most important are the waiting lists. Another equally important factor that may reduce accessibility is the uneven geographical distribution of services. An important factor that can reduce accessibility is the health system itself since only modern health services in developed countries are characterized by universal coverage and equal access by citizens.

This indicator is a number that shows the average length of waiting lists (in days) for both general and specialized mental health services. This indicator may be referred to as the waiting list for mental health professionals, that is the psychiatrist, psychologist, social worker, mental health nurse, psychotherapist, counselor, or occupational therapist.

The goal of the performance is to eliminate waiting lists from the health system. The existence of a waiting list leads to a loss of public confidence in the health system. It leads beneficiaries not to use the services to which they are entitled, and to purchase services from private providers outside the health system, by making direct out-of-pocket payments or by buying private insurance contracts.

#### 3.2.3. Waiting Days for Admission to the Clinic (Waiting Lists)

Psychiatric clinics can be roughly divided into two categories, depending on the patient admission procedure. One category is emergency psychiatry clinics that treat a patient with a temporary safe, compulsory care order. Usually, these clinics are in psychiatric hospitals and rarely in general hospitals. The other category is the psychiatric clinics that treat patients only on a voluntary basis, at the direct request of the patient and after the patient is first informed and has signed the consent form. Second-category psychiatric clinics are usually located in general hospitals and rarely in psychiatric hospitals. There are also mixed clinics where cases are treated with compulsory or voluntary hospitalization. There are no waiting lists for mandatory hospitalizations. For voluntary care, there may be long waiting lists sometimes. Long-term waiting lists usually refer to cases of long-term voluntary care, such as treatment for personality disorders or even more often to drug and alcohol detoxification and rehabilitation centers.

This is a structure indicator. The indicator is the average number of days a patient has to wait before being admitted to the clinic. Waiting lists are due to under-staffing and result in reduced accessibility for beneficiaries. The lack of transparency in waiting lists can conceal corruption, i.e., the use of public power for personal gain.

The goal is to eliminate waiting lists to ensure equal access for beneficiaries to treatment.

#### 3.2.4. Percentage of the Population Having Access to the Health System

This is a structural indicator of the system that concerns citizens’ accessibility to the system. Ideally, health systems should ensure universal coverage and equal access to the citizens of the country, but also to all EU citizens, on the basis of the European acquis on cross-border health between the Member States. Indicatively, it is reported that in Cyprus [11,15] and Greece [16,17] the entire population now has access to the country’s health services. However, there are residents (legal or illegal) who do not have access to the services because they are not citizens (they do not have citizenship/nationality). Special arrangements should be made for these categories to have access to mental health services for those suffering from acute or chronic mental disorders and, in particular, for those suffering from chronic substance dependence. Denial of access of this population to mental health services results in exacerbation of social problems and bio-psychosocial dysfunction for the patient.

The indicator is a fraction where the nominator is the number of residents of the country having access to the country’s mental health services, and the denominator is the total number of residents of the country. The health system access must include legal residents and non-legal residents as well as temporary residents, as all of these people are affected and affect public health. Certainly, there are statistical difficulties in measuring the population who are not citizens of the country as they are a hidden population that is not recorded in the population censuses.

The benchmark and performance target is full (100%) population access to mental health services. This seems to have been achieved in some countries for their citizens (e.g., in Cyprus and Greece). However, the objective of achieving this must be the accessibility to services for both EU citizens as well as for illegal immigrants/refugees. Whether or not some of them may be deported in the future, the country of residence must provide them with health services for the time they are on their territory, in the context of patients’ rights to access health services regardless of whether legal cases are pending against them. This is due to the fact that the right of patients to health refers to patients in the territory of the country, and not only to the subpopulation of patients who are citizens of the country.

### 3.3. Acceptability Criterion or Acceptability Dimension. Indicators of Acceptability

#### 3.3.1. Average Patient Satisfaction Rating

Patients themselves, as service users and customers, evaluate the quality of service provided by the provider. Mentally ill patients may not have the full critical ability to evaluate the service provided, especially if they suffer from acute psychosis, without or with reduced insight, and are treated with a temporary compulsory safe care order. For this reason, mental patients respond to patient satisfaction questionnaires at the end of their hospitalization, and in particular, when their critical capacity has been restored, and questions can be sufficiently understood.

This is an outcome indicator. The indicator is the average of the ratings of patient responses to patient satisfaction questionnaires/scales.

The average value of responses can be assessed in two ways. Firstly, by comparing directly with the responses given by patients treated in other relevant centers in the country or abroad. Second, by comparing the responses of patients in the same center over time. The analysis of the answers reveals and identifies the weaknesses of the center/service being evaluated, and improvements are planned while comparing this year’s answers with those of previous years. The index evaluates whether this service improves over time. Obviously, patients’ evaluation questionnaires should be systematically answered over time by all patients who are discharged and not confined to a specific study of a specific time period. Such questionnaires are integrated into modern comprehensive assessment programs (360 degrees), where everyone involved in health services is evaluated by everyone.

#### 3.3.2. Recording Patient Experiences

Recording patients’ experiences in free-text formats is important for quality assessment and for designing improvement interventions. The recording should be done on a systematic basis over time. Registration should be anonymous and should be done by all patients upon discharge from the clinic so that there is no conflict of interest. The appropriate time to respond in writing to the patient is to ask about their experience on the day of removal, immediately after being discharged, but before the patient left the hospital.

It is an indicator of the qualitative evaluation of the processes and the outcome. It is a qualitative record of the experience the patient has experienced while in the hospital or in general in the treatment of the patient.

Reduction in the number of negative patient experiences compared to the previous year in the same clinic can be used as a performance target.

#### 3.3.3. Patients’ Rights to Information and Protection of Their Personal Data

This is a process indicator that refers to the mental patient’s access to their medical information. The patient alone has the right to know all the information that is related to the patient’s health. The medical record belongs to the patient. The above are also regulated by the General Data Protection Regulation (General Data Protection Rules, GDPR) [18,19]. Member States have incorporated these aspects of the General Data Protection Regulation into separate legislation, such as the Electronic Health Act of the Republic of Cyprus [20].

The index is scored on a questionnaire that includes questions about the patient’s satisfaction from the mental health service, regarding the personal data management, and the extent to which the patient is aware of their rights with regard to their personal sensitive data.

The performance objective is to improve the results compared to the previous year’s results of the same service. This indicates an improved, over time, communication, and information of the patients regarding their rights, but also an adaptation of the mental health service to the General Data Protection Regulation.

#### 3.3.4. Patient Rights to Health Services

Beneficiaries of health systems often do not fully know their rights and obligations within the health system. Accessibility often differs between different services in the same health system, but the obligations of the beneficiaries, as well as any financial obligations (co-payments), also vary. Accessibility is also affected by the beneficiary’s educational level, financial status, accessibility to the Internet, and distance to the nearest medical service.

This is a process indicator. The indicator is a mean score of patients’ responses to a questionnaire with questions about the basic rights of patients, as provided to beneficiaries by the health system, but also as provided for by the legislation of the Member State and the European acquis.

The performance target is to improve the index compared to the previous year’s average patient rating in the same questionnaire.

#### 3.3.5. Number of Patients Leaving the Clinic with a Discharge Note

Patients hospitalized in psychiatric hospitals or in general hospital’s psychiatric clinics are admitted, on a voluntary or on a compulsory basis, for hospitalization. They may be hospitalized on a voluntary basis have become aware of the need for treatment (insight) or maybe hospitalized on a mandatory basis without being aware of the need for treatment (no insight). In the latter case, the patient is treated under a court order for a temporary mandatory safe hospitalization and dismissed when symptoms have subsided, and the patient’s critical mental capacity has returned. Consequently, both of the above categories of patients (with some exceptions) have recovered their mental capacity as a result of the treatment and are entitled to be informed in writing of their health status, receiving a notice upon completion of their treatment and discharge from the clinic.

This is a process indicator, which relates to patients’ access to information. The index is a fraction, where the nominator is the number of patients discharged from the clinic with a discharge note, and the denominator is the total number of patients discharged from the psychiatric clinic.

The performance target is all of the patients to receive an information note, the day of their discharge from the clinic.

#### 3.3.6. Number of Patients Using the Suggestion/Complaint Box

In order to assess and improve the quality of care provided, mental health services can easily install a box of suggestions and/or complaints in any inpatient and outpatient setting, e.g., outpatient clinic. Subsequently, psychiatrists encourage patients (inpatient and outpatient) to write a letter or fill out a form with their suggestions for improving the service (or their complaints) and adding it to the box. Patients’ notes can be a valuable tool for qualitative-rather than quantitative-assessment of the quality of each mental health facility because they contain descriptions of the experiences patients have had in their treatment in the particular clinical setting.

This is a process indicator. This indicator is a fraction: The nominator is the number of patients who used the suggestion/complaint box during a year, and the denominator is the total number of patients discharged from the clinic during the same time period.

There is no published benchmark for this indicator because it is a quality evaluation indicator. Letters/notes describing patients’ experiences are useful for evaluating the quality of care. An increase in the number of complaints per year may indicate a decline in quality due to under-staffing or under-financing.

Alternatively, all clinics (inpatient and outpatient) could suggest to all patients on the day of discharge from the clinic to write a summary or fill in a form/questionnaire (in print or electronic form), revealing their treatment experiences. In some hospitals/clinics, this form/questionnaire is considered a patient’s obligation. Other hospitals could choose to motivate the patient to complete the form/questionnaire. The incentive could be economical, in the form of a reduction in co-payments, for example, to medications that patients may receive from the hospital pharmacy prior to discharge.

### 3.4. Competence Criterion or Competence Dimension. Indicators of Competence

#### 3.4.1. Lifelong Learning Program for Mental Health Professionals

In modern mental health systems, lifelong learning programs for mental health professionals are essential for maintaining the quality of services, productivity, and satisfaction of patients and employees.

This is a structure indicator. The indicator is a fraction where the numerator is the number of mental health professionals in a lifelong training program, and the denominator is the total of mental health professionals working in the same institution.

The goal is to increase the index year after year, with the ultimate goal of ensuring that all employees in the organization enjoy lifelong learning.

#### 3.4.2. Lifelong Learning Program with Quantification of Training for Mental Health Professionals

Scientific developments in the field of mental health are running at a rapid pace, while the environment is changing. Therefore, there is a need for continuous modification of the bio-psychosocial diagnostic and therapeutic interventions that a well-trained health professional should provide. To this end, health professionals must have lifelong training. It is good practice for the training to be quantified in units to ensure the required training on an annual basis.

This is a process indicator. The index is a fraction where the numerator is the number of specific specialty mental health practitioners who follow a Lifelong Curriculum that is quantifying in units, and the denominator is the total number of health professionals of the same specialty.

The aim is to increase the index score, year by year, with the ultimate goal of ensuring that all mental health professionals follow a qualitative and quantifiable educational system with training units.

### 3.5. Effectiveness Criterion or Effectiveness Dimension. Indicators of Effectiveness

#### 3.5.1. Percentage of Patients Who Have Improved as a Result of Treatment

Patients are usually admitted to mental health clinics during an acute mental disorder or during exacerbation of a chronic mental disorder. Patients are discharged from the clinic after receiving appropriate treatment and improvement. Appropriate therapeutic interventions are usually a combination of biological interventions (psychotropic drugs), non-medication interventions (psychotherapy, counseling), and social interventions. The “percentage of patients who have improved” is a useful and, therefore, common and popular indicator of the quality of mental health service [10].

This is an outcome indicator. The index is defined as the fraction the nominator of which is the number of patients identified in the discharge note as “improved”, and the denominator is the total number of patients discharged from the same mental health clinic over the same time period, usually over a period of one year. Patient improvement may refer to the initial symptoms present at the day of admission, but may also refer to the patient’s psychosocial functioning as assessed by a clinical examination or to a scale such as the Global Assessment of Functioning (GAF) [21,22].

The proportion of patients who have improved may vary from clinic to clinic, depending on the type of diagnosis and the severity of the cases. However, most patients are expected to show improvement as a result of the treatment provided in the clinic. It is recommended that all clinics publish the “percentage of patients showing improvement” index so that quality can be monitored and compared with other clinics over time. A common reason for the patient not improving may be the patient’s early voluntary discharge from the clinic despite the psychiatrist’s dissenting opinion that the patient has to remain in the clinic until completion of treatment. The performance target varies depending on the severity of the cases being treated by each clinic. However, the indicator is particularly useful for improving the clinic through comparisons over time.

#### 3.5.2. Extra Mortality in Patients with Schizophrenia Overall and by Sex

Patients with schizophrenia have a lower life expectancy compared to the general population. This decline is a result of many factors, such as cognitive decline, poverty, lack of social support, reduced ability to co-operate in treatments for physical ailments (e.g., diabetes, hypertension), and an increased likelihood of suicide.

This is an outcome indicator. Extra mortality in patients diagnosed with schizophrenia is defined as a fraction where the total mortality of schizophrenia patients is the numerator, and the overall mortality of the general population is the denominator. Both the numerator and the denominator include only patients aged 15 to 74 years, according to OECD recommendations [23].

This indicator should be presented for men, women, and the general population. A number of benchmarks have been published from time to time [23]. According to OECD published data, Latvia has the lowest rate, 1.7 for men and 2.4 for women. The highest index is reported by Finland and is 6.6 for men and 6.1 for women. Only Finland and Denmark report higher rates for men, while other OECD members report higher rates for women than men.

#### 3.5.3. Extra Mortality in Patients with Bipolar Disorder Overall and by Sex

Patients with bipolar disorder have increased mortality and reduced life expectancy. This is the result of many factors, including an increased risk of suicide compared to the general population, increased correlation with substance use disorders, reduced income due to loss of work, obesity, and cognitive impairment and brain atrophy (mainly of the white matter bundles) as a consequence of the natural course of the disease.

This is an outcome indicator. The index “increased mortality in patients with bipolar disorder” is a fraction, with numerator the total mortality in patients with bipolar disorder (type-1 or type-2), and denominator the number of patients with general mortality in the general population. The numerator, as well as the denominator, includes only ages 15 to 74, according to OECD guidelines, as mortality in the rest of the age range is not affected by bipolar disorder [23].

This indicator should be presented for men, women, and the general population. Israel has published the lowest values, which are 2.8 for men and 2.6 for women. The OECD has published benchmarks according to which: Finland has the highest values for women, i.e., 5.2. Korea has the highest values for men, i.e., 3.7. All countries, with the exception of Israel, have a higher mortality rate for bipolar disorder in women than in men [23].

#### 3.5.4. Number of Psychiatric Beds per 100,000 Inhabitants

This is an indication of the state of mental health in a country. A very small number of psychiatric beds in a country can mean that the country does not give enough money for mental health; that is, mental health is not in the country’s priorities. On the other hand, too many beds may mean that the country has not undergone sufficient psychiatric reform/deinstitutionalization, and/or that there is not a sufficient number of community psychiatric services.

This is a structure indicator. The index is a fraction where the number is the number of psychiatric beds in the country, and the number is 100,000. The index is published on Eurostat on an annual basis by all Member States.

The benchmark is the average number of psychiatric beds in the Member States. The performance target is to bring health systems closer to the European average in terms of the number of psychiatric beds in each country. The average number of psychiatric beds in Europe of 28 Member States is 69 beds per 100,000 inhabitants [24]. Indicatively, psychiatric beds in Cyprus are only 21/100,000 inhabitants, in Greece 74/100,000, in Belgium 136/100,000, in Germany 128/100,000, and in France 84/100,000, according to published data in the European Union Statistical Office (Eurostat) [24]. Monitoring the course of the number of psychiatric beds in each country can yield useful conclusions about the mental health of each member country. An extremely low number of psychiatric beds indicates underemployment and lack of funding in mental health, and that mental health is not a priority for that country. On the other hand, an extremely high number of beds may indicate that psychiatric reform in some countries has not progressed sufficiently, although mental health policy in countries with high scientific tradition and mental health research (number of publications in peer review psychiatry journals of high impact factor) is very important to be taken into account.

#### 3.5.5. Inpatient Suicides

The suicide of a patient while hospitalized in a psychiatric hospital is a very rare event. However, it is the worst that can happen.

This is an outcome indicator. The Inpatient Suicide Index is a fraction, in which the numerator is the number of inpatient patients who committed suicide during their stay in the facility (general hospital, psychiatric hospital, prison hospital, prison setting) and denominator is the number of patients in the respective unit structure that have a primary or secondary diagnosis of a mental disorder.

Inpatient suicide is a very rare event, so the performance target should be zero. The benchmark is close to zero in most countries. Indicatively, the Czech Republic reported the lowest rates with 0.0% and Spain with 0.02% of inpatient suicides in a 2013 study. Estonia gave the highest rate of 0.025% for the same year, which is, nevertheless, very low [23].

#### 3.5.6. Suicides of Patients Hospitalized in a Psychiatric Facility in the Last Month and Previous Year

This is an indicator that is particularly characteristic of the quality of mental health services provided in each country. It is also characteristic of the continuity of care provided to potentially suicidal patients who have been discharged from mental health clinics of any type.

This is an outcome indicator. This indicator is a fraction where the numerator is the number of suicides recorded in the last month and the last 12 months, and the denominator is the number of patients discharged with primary or secondary psychiatric diagnoses during the past month and the last 12 months respectively. For each case, the follow-up period begins the day of the discharge from the inpatient clinic.

For both of these indicators, the UK is considered the benchmark country, with less than 0.2 suicides per 100 discharged patients each year, or less than 0.1% suicide rate for the first month after being discharged from a psychiatric hospital. Slovenia has the highest value in the suicide rate for the first year after the discharge, with more than 0.8% for the first year or more than 0.3 for the first month after the discharge [23].

#### 3.5.7. Suicides in the Country Last Year

This is an outcome measure that is a powerful predictor of the level of mental health of each country’s population. The indicator is not stable but is influenced by environmental factors. The index is heavily influenced by the country’s economic level, with prices rising during the economic crisis [25]. In Greece, suicide attempts and suicides increased significantly during the financial crisis [26].

The index is a fraction where the numerator is the number of suicides in the country in one year per 100,000 population, and the denominator is 100,000. In addition to the overall index, it is informative to present the index for men and women separately.

Greece and Cyprus are benchmarks for this indicator, with the lowest value of five suicides per 100,000 population per country. The indicator is considered typical for the level of Psychiatry and Mental Health in each country. Lithuania has the highest rate in the index, with 30 suicides per 100,000 population per year. Liechtenstein, Cyprus, Greece, and Italy have the lowest rates with 2,4,5,6 suicides per 100,000 population per year respectively. For the EU Member States, the average is 11 suicides per 100,000 population per year [27]. However, overall for the WHO European Region, which includes the poorest European states outside the EU (a total of 53 countries including the EU-28 Member States), suicides are 21.2 per 100,000 population per year [28].

#### 3.5.8. Percentage of Patients Who Completed the Addiction Treatment Program

The indicator relates to voluntary inpatient treatments in psychiatric clinics or detox/rehab clinics. Any patient who enters such a treatment setting can either complete the treatment and be discharged, or interrupt and leave the clinic before completing despite the physician’s contrary opinion. When many patients discontinue voluntary treatment, this may be indicative of (1) lack of education by health professionals, (2) excessive rigor of the program, (3) excessive severity of the disease of patients admitted to the clinic (severe drug/alcohol use disorder), (4) lack of human resources, staff are not numerous enough for quality hospitalization/monitoring/intervention.

This is a process indicator. The index is a fraction where the number of patients who completed the treatment program is the numerator, and the denominator is the total number of admissions to the same clinic over the same period of one year.

The index can be used for comparison between similar clinics of the same or other hospitals. However, it is more useful for monitoring the course of the same clinic over time, comparing this year with the previous year’s score. In international literature, some authors prefer to calculate the dropout rate. The performance target is to improve the index against last year’s values in the same clinic.

#### 3.5.9. Percentage of Substance Abusers Who Have Reduced Consumption as a Consequence of Treatment

The modern approach to the treatment of substance use disorders (detoxification, rehabilitation, harm reduction) does not only include treatments that are oriented towards complete abstinence. Previously, treatment programs were always abstinence oriented, and these approaches were commonly known as “abstinence-oriented therapeutic communities”. In the modern era, the “Harm Reduction” approach is popular, where complete abstinence is desirable but not necessary for the achievement of treatment. Treatment is defined as an increase in bio-psychosocial functionality, even if the patient remains a sporadic user of the legal or illegal substance. Increased bio-psychosocial functionality means rehabilitation of physical illnesses related to use (e.g., infections, liver disease, etc.), rehabilitation of psychological stability (treatment of withdrawal syndrome, depression, etc.), social rehabilitation (back to home, family, work, interpersonal relationships, no more unlawful actions and issues with law, police, courts, prisons, etc.). In order to evaluate the effectiveness of therapeutic interventions, conservative endpoints such as days until relapse, which refer to days of abstinence after leaving the program, were previously selected. Additionally, “the percentage of patients who completed the treatment program” is a criterion referring to the percentage of patients who do not discontinue treatment before it is completed. More modern and less conservative evaluation indicators of therapeutic interventions used in the harm reduction approach are “average daily consumption”, as well as the number of “days where daily consumption exceeded” two international alcohol units or another threshold set by the psychiatrist/researcher.

This is an outcome indicator. The index is a fraction where the number of patients who have restricted consumption after the treatment program is the numerator, and the denominator is the number of patients who participated in that treatment program.

The performance target is the index value getting closer to the unit through continuous program improvements. The index can be used as a program self-improvement tool but can also be used to compare different programs when these programs have a therapeutic goal of reducing consumption.

#### 3.5.10. Percentage of Patients Readmitted in One Month

The indicator refers to the phenomenon of “revolving door.” Patients with coexisting severe psychosocial problems and a lack of supportive environment return to the clinic soon after their discharge. Some of these patients suffer from severe chronic recurrent diseases and frequent readmission may indicate inadequate structure for their medical care, for example, a patient with a severe personality disorder hospitalized in a general hospital’s mental health clinic.

This is an outcome indicator. The index is a fraction where the numerator is the number of patients readmitted within the first month after their release, and the denominator denotes the total number of patients dismissed from the psychiatric clinic within the same time period.

The goal is the avoidance (or decrease the number) of readmissions to a psychiatric clinic within the first-month post-discharge, but instead to continue patient’s treatment on an outpatient basis. This presupposes the existence or establishment of appropriate outpatient structures for community psychiatry and day hospital for mental patients.

#### 3.5.11. Percentage of Patients Who Continued Relapse Prevention Therapy after Detoxification

Treatments for drug dependencies, whether they are drug dependencies or behavioral dependencies, consist of two major distinct phases. The first phase is detoxification; that is, the treatment of withdrawal syndrome, achieved in combination with pharmacotherapy and psychotherapeutic interventions. The second phase is relapse prevention, which lasts much longer and is also done with a combination of pharmacotherapy and psychotherapeutic interventions. Often the first phase (detoxification) takes place in the inpatient clinic, followed by the second phase (relapse prevention), which takes place in the outpatient clinic.

This is an outcome indicator. The indicator is the percentage of patients who continue into the second phase after the first phase is completed.

The goal of the performance is for all patients to continue and complete the second phase. The reason many patients do not go on the relapse prevention phase is that by the end of the first phase, the patient is already abstaining from alcohol while the symptomology of withdrawal syndrome has completely subsided. This gives the patient the illusion that they have completely regained control and do not need further treatment. The patients who do not enter the relapse prevention program may have increased risk for relapse.

#### 3.5.12. Number of Abstinence Days after Discharge from the Clinic (Days until Relapse)

Substance use disorders, such as alcohol and illicit drug dependence, are chronic relapsing mental disorders and as such, are classified in the Psychiatric Disorders Classification Systems, such as DSM-5 of the American Psychiatric Association [29] and ICD-11 [30] of the World Health Organization. Since these are chronic recurrent disorders, this means they have relapses and remissions. Consequently, relapses should not be interpreted as a failure of treatment, but there are embedded in the natural course of the disease. The purpose of treatment is to reduce the number, frequency, duration, and intensity of relapses.

This is an outcome indicator. The indicator is the average number of days that the patient remains in abstinence from alcohol or the illicit recreational substance of dependence after the completion of a specific treatment plan. The program may be for example, a short-term detoxification program or a long-term inpatient therapy program, e.g. in a therapeutic community setting.

There is no published benchmark. The index can be used to compare similar programs with one another, as well as to document treatment program improvement over time. The index can also be used as a clinical evaluation endpoint in research protocols for the evaluation of therapeutic interventions over time (randomized clinical trials, randomized control trials, RCTs). The indicator is about abstinence-oriented therapeutic interventions or treatment programs, and not so much the modern harm reduction interventions.

#### 3.5.13. Number of Days with Consumption More Than Two Units of Alcohol (Iu, International Units)

In the treatment of addictions, complete abstinence has been, in the past, the only treatment option. In recent years, the therapeutic option of controlled consumption has also been added. This option is mainly recommended for patients that are suffering from addiction on legal substances such as alcohol, and not for patients addicted to illegal substances. However, in legal and illegal substances of abuse, the harm is dose dependent, which is high, and uncontrolled consumption is certainly more harmful than low controlled consumption. This also applies to behavioral dependencies such as the gambling use disorder, where mildly controlled betting may be less harmful than uncontrollable. Consequently, the reduction in consumption is accompanied by a reduction of harm, to both substance dependencies and behavioral dependencies.

This is an outcome indicator. The indicator refers to alcohol, where there is a quantified dose. In particular, an international unit (IU) is equivalent to a drink containing 12 grams of ethanol, that is, about a small beer, a glass of wine, or a glass of whiskey. It is an outcome measure where it evaluates patients completing a treatment plan for addiction, with a therapeutic goal of controlled use. The indicator is a fraction, where the numerator is the average number of days that patients consume more than two units of alcohol, and the denominator is the sum of the days of patients completing the treatment program under study.

There is no published benchmark. The indicator is useful for evaluating treatment programs aimed at reducing consumption, i.e., controlled use. These are harm reduction programs and not complete abstinence programs. The index is also useful as an endpoint in research programs to reduce the harm from alcohol use.

#### 3.5.14. The Average Consumption of Alcohol or of Other Recreational Substance of Abuse after Discharge from the Clinic

Addiction is a chronic relapsing mental disorder, which means that relapses are included in the natural course of the disease. Many patients that are suffering from drug use disorder are starting their treatment under genuine internal motivation. Others, however, have only external motivation. Therefore, efforts are made by the therapists during the treatment to transform this external motivation into internal motivation. Some patients do not want, are not ready, or cannot discontinue the drug and/or alcohol use, so reducing consumption is a temporary harm reduction intervention, as the harm is dose dependent.

This is an outcome indicator. The indicator is the average alcohol consumption per day for the following year after completion of a treatment program. Accordingly, the indicator can also be used for illegal euphoric substances.

There is no published benchmark. The indicator is important for evaluating treatment plans and/or individual therapeutic interventions. It can also be used in harm reduction research or drug evaluation protocols aimed at reduced alcohol or drug consumption.

### 3.6. The Criterion of Therapeutic Continuity or Therapeutic Continuity Dimension. Therapeutic Continuity Indicators

#### 3.6.1. Percentage of Patients Continuing an Outpatient Follow-Up Program after Completion of the Inpatient Treatment Program

Detoxification and rehabilitation therapies from legal and illegal additives often contain two distinct phases of treatment. The first stage—usually within the clinic—is detoxification, that is to say, the discontinuation of the addictive substance and the prevention/treatment of impending withdrawal syndrome. In the second phase—usually on an external basis—it is prevented from being a relapse, by managing/treating the strong/irrepressible desire (craving) for drug/alcohol use. Many patients abandoned the program after completing the first phase of treatment, mistakenly assuming that they have gained control of consumption, and therefore, no further treatment would be needed to prevent a recurrence.

This is an outcome indicator. The indicator is a fraction, where the numerator is the number of patients continuing on an outpatient program after completion of detoxification, and the denominator is the number of patients who have started detoxification therapy.

The goal of performance for all of the patients is, after completing the internal phase, to continue to their outpatient relapse prevention program.

#### 3.6.2. Percentage of Patients Attending the First Follow-Up Appointment as an Outpatient after Being Discharged from the Psychiatric Clinic

In psychiatry, as a general rule, when a patient completes a period of hospitalization and is discharged from the clinic, the patient continues to be an outpatient in the outpatient clinic. The occurrence of the first appointment is indicative of the patient’s compliance/co-operation or otherwise adherence with the treatment, which is related to the patient’s mental state at the time of discharge, as well as to the therapeutic relationship established between the patient and the therapist during the internal phase of treatment.

This is an outcome indicator. The index is a fraction where the numerator is the number of dismissed patients who appeared at the first scheduled appointment after discharge, and the denominator is the number of patients who appeared together with those who did not appear at the first scheduled appointment.

Generally, for patients with mental health problems who were given their first appointment one week after completion of internal therapy and discharged from the clinic, a satisfactory performance target is the appearance of 80% of patients, according to published data [31]. 

#### 3.6.3. Percentage of Patients Attending the First Follow-Up Appointment after Discharge from an Inpatient Clinic for Treatment of Substance Use Disorder

Addiction/dependence or, more precisely, Substance Use Disorder is a chronic, relapsing and remitting mental disorder. Usually, this patient population suffers from co-morbidities, mainly with personality disorders, and this is what diminishes their commitment and co-operation in treatment.

This is an outcome indicator. The index is a fraction where the numerator is the number of patients who completed the detoxification/rehabilitation program and then discharged and received their first appointment as outpatients. The denominator is the number of discharged patients who attended together with those who did not attend the first scheduled appointment as outpatients.

For the general population of patients with mental health disorders who have their first re-evaluation appointment one week after discharge, the performance target is 59% according to published data [31]. This compliance rate for patients with substance use disorder is significantly lower than the general population of patients with mental disorders who come to the first appointment after dismissal, which is 82.4%, according to the findings of the same study. The large difference may be due to the reduced compliance/co-operation of the subgroup of mentally ill patients with substance use disorder, due to co-morbidities with personality disorders.

#### 3.6.4. Percentage of Patients Who Attended the Next Two Appointments after Being Discharged from Hospital

The phenomenon of patients losing their appointments at an outpatient clinic is occasionally observed. However, it is not observed with the same frequency in all areas of the General Health System. Lost appointments show difficulty in this structure for maintaining the therapeutic continuity, which is necessary to improve prognosis.

This is an outcome indicator. The index is a fraction where the numerator is the number of cases that came to the next two appointments after discharged from the hospital. The denominator is the total number of discharged patients from the clinic at the same time period.

There is no published benchmark. The purpose of the indicator is to improve the clinic itself. A low score means that the clinic has difficulties maintaining patients for long-term follow-up, that is, maintaining therapeutic continuity. The causes need to be sought and corrected. In searching for the causes of missed appointments, it is important to ask the patients’ opinions by using questionnaires, oral interviews, or written free text. Patients should always be taken into account.

#### 3.6.5. Number of Patients Attending Two Consecutive Appointments

The continuity of therapeutic care is an important indicator of the quality of the system. It helps to complete treatment, reduce complications, prevent relapse, and improve the prognosis of patients with mental disorders.

This is an outcome indicator. The index is a fraction where the numerator is the number of patients with a chronic mental disorder who came to two consecutive appointments, and the denominator is the total number of patients.

The goal is to increase the number of patients who do not miss their scheduled appointments, year after year, that is, they have years of constant monitoring in the health system.

#### 3.6.6. Number of Referrals

The number of referrals indicates continuity of care (therapeutic continuity) but also indicates the extent to which a mental health service operates interdisciplinary.

This is a process indicator. The index is a fraction where the numerator is the number of patients who received a referral from a physician on the day of discharge from the clinic. The denominator is the total number of patients dismissed from the clinic during the same period.

There is no published performance target for the referral rate, but the index can be used by clinical managers as a tool to improve communication between medical specialties and between different disciplines, as well as an indicator of ongoing clinical evaluation.

### 3.7. Efficiency Criterion or Efficiency Dimension. Efficiency Indicators

#### 3.7.1. Number of Mental Health Professionals per 100,000 Population

The number of healthcare professionals per 100,000 population, mainly psychiatrists per 100,000 population and mental health nurses per 100,000 population, varies between countries, and in many cases, this is indicative of the population’s access to mental health services, which may also affect the quality of mental health services.

It is an indicator of the structure of the country’s mental health services. The index is a fraction of the number of mental health professionals (e.g., psychiatrists) per 100,000 inhabitants.

Specifically for psychiatrists, according to the WHO regarding the WHO European region, there are 13 psychiatrists per 100,000 inhabitants, while in the European Union of 28 Member States, the number is much higher [32]. The performance target for each Member State is the convergence index to the European average. The European average is 43.5 mental health professionals per 100,000 inhabitants. Indicatively low in the index is Africa and South Asia, where the WHO lists only 1.4 and 4.8 mental health professionals per 100,000 population, respectively [33]. Specifically, in the WHO European Region, in every 100 mental health professionals are included ten psychiatrists, one Child and Adolescent psychiatrist, three physicians of other specialties, 23 mental health nurses, five psychologists, one social worker, half of an occupational therapist and half of a speech therapist (that is, one speech therapist per 200,000 population) [28].

#### 3.7.2. Percentage of the Workforce in the Country That Is Working in the Health System

In the EU Member States, the health system is a major employer: Much of the country’s workforce is employed by public or private health services. Comparing the staffing of different health systems can lead to different conclusions, especially when comparing public health indicators. The low score on this indicator may explain the low accessibility of beneficiaries in certain areas/services of the health system.

This is a structure indicator. The indicator is a fraction where the numerator is the number of mental health professionals working in the health system, and the denominator is the total workforce in the country.

The performance target is intended to be close to the European average in each Member State. The deviation from the European average may indicate the need for adjustments, either by funding, an increase of human resources or productivity. The WHO has published data that in Europe, 50 health professionals per 100,000 population [28] work in the field of mental health. According to the same report, specifically for the list of rich countries as defined by the World Bank-including Greece and Cyprus-there are currently employed 71.7 mental health professionals per 100,000 inhabitants [28].

#### 3.7.3. Number of Patients per Physician per Day, in the Outpatient Clinic

Psychiatric evaluation, counseling, and intervention require the formulation of a therapeutic relationship and quality communication between the psychiatrist and the patient at each session, and this is a time-consuming task that cannot be done in a hurry. Time is a limiting factor in the number of patients that each psychiatrist can see per day of an outpatient clinic. When there are many patients per psychiatrist per day of outpatient care, the time available per patient is less, resulting in a decline in the quality of service provided.

This is a structure indicator. The index is a fraction where the numerator is the number of outpatients that the psychiatrist has served in clinic during the year, and the denominator denotes the number of full working days in an outpatient clinic.

The performance target is that the number of patients is about 10 per day in the outpatient clinic and certainly not more than 20, meaning each session lasts about 40 minutes, with short breaks between sessions. For Cyprus, the Union of Medical Doctors of the Public Sector (PASYKI) has set the maximum number of patients that a specialist should not exceed in one full clinical working day, in the secondary specialist care outpatient clinic, in 20 patients per day. However, difficult cases within specialties such as Psychiatry contribute to a rate greater than one patient per hour.

#### 3.7.4. The Average Length of Hospitalization

Patients with the same diagnosis in different clinics need a different (greater or lesser) number of days of hospitalization until their hospitalization is complete. This is related to staffing, experience, clinical workload, availability of labs and imaging laboratories, and many other parameters related to the availability of logistical and human resources.

This is an outcome indicator. The numerator is the total number of days of hospitalization per clinic per year, and the denominator is the total number of patients hospitalized in the clinic over the same time period.

The purpose is quick, but not hasty, case management. For this reason, the index should be used to compare similar clinics and similar cases, for example, by using of Diagnosis Related Groups (DRGs).

#### 3.7.5. The Average Cost of Hospitalization per Patient per Doctor, Clinic, Field, Hospital

The average cost of hospitalization is an important parameter for financial planning, financial management of the hospital, and its sustainability and sustainable development. The average cost of hospitalization can be calculated per patient, per entry diagnosis, per physician, per clinic, per department and per hospital.

This is an outcome indicator, which is influenced by the days of hospitalization of each case. The total operating cost of the hospital, ward, clinic, is divided by the total days of hospitalization, and the cost per day of hospitalization is calculated in each case. Then the amount is multiplied by the average number of days of hospitalization of patients and thus, the average cost per patient is calculated. The average cost per patient per doctor is also calculated correspondingly since, for patients with the same diagnosis, the days of hospitalization differ between different physicians but also between different hospitals and between different clinics of the same hospital. The average cost per hospitalization is also calculated per diagnosis/treatment using the Disease-Related Groups (DRGs) method, based on which a specific hospital fee is set for each DRG. The DRGs method is not currently available in all hospitals.

It is not easy to define the benchmark that is a performance target, as costs should be correlated with the outcome of the incidents using an outcome indicator. The index is very useful for comparing hospital costs between hospitals in the same country. However, it can also be used for between-countries comparisons if the cost of hospitalization is corrected for the difference in Gross Domestic Product (GDP) between the two Member States. However, in any health system, the average cost indicator must be calculated, and the clinics, hospitals, and physicians that deviate from this should be mobilized towards convergence.

#### 3.7.6. The Average Cost per Day of Hospitalization per Clinic, Sector, and Hospital

The average cost per patient varies between clinics, departments, and hospitals for a variety of reasons. For the management of patients with the same diagnosis, there can be a great difference in the days of hospitalization but also in the type and cost of examinations performed between different clinics, departments, hospitals. System sustainability and sustainable development are important parameters in all modern health systems, at least as far as the EU Member States are concerned. Therefore, the cost per day of hospitalization is an important parameter.

This is an outcome measure that is the cost of each day of hospitalization. The total cost of the hospital, ward, and clinic is divided by the total days of hospitalization, and the cost per day of hospitalization is calculated in each case. The index is a fraction, where the numerator is the total cost of operating the clinic for one year (salaries, building, medicines, consumables, etc.), and the denominator is the total patient-days of a year of the clinic.

This is a very useful index on the basis of which managers can compare the cost between clinics and between physicians of the same specialty. However, evaluation cannot be done only by cost indicators, but clinical outcomes must be taken into account, together with other epidemiological health indicators. It is not possible to make direct cost comparisons between different clinics and different countries because there are many different factors, payrolls, procedures, diagnoses of cases admitted, therapeutic protocols that determine the days of hospitalization in each case. The indicator is very useful for comparing costs between the same clinics located in different hospitals in the same Member State. For example, a comparison of the cost per day of hospitalization could be performed between the two General Hospitals Psychiatric Clinic in two different cities.

#### 3.7.7. The Average Cost of Medication Consumption per Patient and per Day of Hospitalization, per Physician, Clinic, Field, Hospital

With up-to-date medical folder software included in the Integrated Hospital Information System, the total cost of a drug for the entire hospital per year and then per patient, clinic and/or physician can be calculated. The cost per patient depends largely on the diagnosis, but it also depends on other parameters, such as over-prescribing and choosing expensive prototypes in place of cheap generics. Therefore, the pharmaceutical cost of treating the same cases may vary between different hospitals and/or different doctors.

This is an outcome indicator. The indicator is the average cost of medication consumption per patient, doctor, clinic, hospital sector. To calculate it, we first calculate the total pharmaceutical cost of each hospital, clinic, department, physician, using a suitable filter from the software. We then divide by the total number of patients or days of hospitalization of patients treated by the respective hospital, clinic, field, physician, and thus the average cost of medication consumption is calculated.

As with any cost index, one must take into account the prognosis and outcome of patients. It is expected that when a clinic treats many severely ill patients, it will have high pharmaceutical costs per patient and many days of hospitalization per patient. However, the expense indicators are useful for comparing physicians and clinicians of the same specialty, as well as for approximating prescription patterns. As a benchmark it may additionally be used the theoretically expected drug consumption in DDDs, according to the definition of Worldwide Health Organization (WHO): “The Defined Daily Dose (DDD) is the assumed average maintenance dose per day for a drug used for its main indication in adults” [34]. The index is suitable for setting a performance target for the financial planning of each structure, especially for structures limited by global/closed budgets. It can also help clinicians and physicians who are far from average to reduce their pharmaceutical spending.

#### 3.7.8. The Average Cost of Diagnostic Examinations per Physician, Clinic, Field, Hospital

Each hospital has a different ability to perform clinical, laboratory, and biochemical tests, due to differences in equipment and training of human resources. Some doctors write more or fewer tests than others, especially in health systems where there are no protocols to determine which specialties can order each laboratory diagnostic test. On the other hand, many times, whether or not a test is performed is limited by the patient’s private security plan, especially in countries with a multi-insurance health system.

This is a process indicator. The indicator is a fraction where the numerator is the total number of diagnostic tests, and the denominator is the number of patients that can refer to the whole clinic, outpatient clinic, field, physician, hospital. The results of the indicator can be presented in all ways to allow comparisons between clinics, doctors, and hospitals, but also to monitor the course of increases or decreases in costs each year.

There is no internationally accepted benchmark due to the large differences in the cost of these services between the Member States. A comparison could be made, between the Member States, after weighting of the data, for example, on the basis of GDP per capita of each country. The index can be more easily used for within-hospital and between-hospitals comparisons.

#### 3.7.9. Cost per Diagnosis Related Group (DRG)

Diagnosis Related Groups (DRGs), have been developed at Yale University and were primarily implemented in the USA and in particular in the Medicare health subsystem, but were quickly transmitted and implemented (same or modified) in many other countries including Greece. The rationale is that each diagnosis based on the ICD-10 classification system corresponds to a specific DRG, which in turn corresponds to standardized specific costs for the hospital that has been calculated and determined in advance. The hospital for each hospitalization of the patient will be compensated on the basis of the patient’s DRGs, as shown by the diagnosis referred to the patient’s discharge note. The doctor sets the diagnosis by using ICD-10 or ICD-11 codes, and the accounting software corresponds the ICD code to the appropriate DRG code, on the basis of correspondence tables. For each DRG, the hospital accounting office will receive the corresponding compensation from the relevant funding source (e.g., Ministry of Health, an insurance company, insurance agency, etc.).

This is a process indicator that is indicative of the economic organization of the health system. The indicator receives YES/NO values indicating whether the DRGs system is applied to the particular health system or hospital or clinic. If the indicator receives a YES value, further quantification of the estimate is considered useful, i.e., the percentage of the hospital funding that is made through DRGs payments.

The most important DRGs for psychiatry-which are a benchmark-are those of Psychosis, that is, the funding of the hospital that uses DRGs for the care and treatment of patients with a chronic psychotic disorder (e.g., schizophrenia).

#### 3.7.10. Cost per Current Procedural Terminology

The existence and employment of the Current Procedural Terminology (CPT) in the health system, for specific and coded terminology, is very useful for storing and communicating information about medical services and procedures. In particular, this process is useful for administrative, financial, and analytical reasons. The CPT is, therefore, a common language that contributes to mutual understanding between physicians, patients, insurance companies (both in a mono-insurance and multi-insurance environment) and the payers of the services provided [35].

For example, in Cyprus, where the Health System (General Health System, GHS) is mono-insurance (single-insurance), all costs go through the Health Insurance Organization (HIO), which is a single-payer mono-insurance organization. HIO recently published the proposed compensation for the medical practice of any specialty under the CPT system [36]. Based on the above model which includes typical examples of medical transactions and fees for each medical specialty, each physician can make assumptions and predictions about their own median income, and the system can calculate median and average income and thus protect and ensure the viability of the health system.

Indicatively, a typical psychiatric visit (with CPT code CY001 and weight of 2 units) together with psychiatric assessment and/or psychiatric counseling (with CPT code 99242 and weight of 2.7 points) of half an hour duration, has a total CPT weight of 3.8 points. At a unit price of 15 euros, this psychiatric session lasting half an hour and weighing 3.8 units is compensated at 3.8 × 15 = 57 euros [36].

This is a process indicator. The Index receives the values YES/NO depending on whether the hospital system in question operates with a CPT algorithm for compensations/payments, and then lists the cost per CPT. This way, cost comparisons can be made between hospitals and health systems.

There is no published performance target because the cost of each medical practice in each country is multifactorial, and it is difficult to make valid cross-country comparisons. However, the indicator is useful for tracking costs over time as well as for planning health system sustainability.

#### 3.7.11. Doctors to Nurses Ratio

The ratio of the number of doctors to the number of nurses is an important indicator of the quality of the clinic. Poor quality may be due to a lack of skilled nurses and, as a result, poor quality of hospitalization or a lack of doctors’ and nurses’ competence. Therefore, poor quality is due to the effort to perform medicine without enough doctors.

This is a structure indicator. The numerator is the number of doctors in the clinic, and the denominator is the number of nurses in the same clinic, hospital, or system.

The published WHO score for this indicator can be set as a performance target, as described [37]. Indicatively, the National Health System (NHS) of the United Kingdom has one psychiatrist for every five mental health nurses. The National Health System of Greece (ESY) has one psychiatrist for every four mental health nurses. In the Mental Health Services of the Public Sector of the General Health System of Cyprus (GHS) there is only one psychiatrist per 14 mental health nurses, while in the Department of Addiction Psychiatry of the Mental Health Services one psychiatrist corresponds to 28 mental health nurses [38].

#### 3.7.12. Doctors per Nurse per Bed in the Hospital

Following the recent financial crisis that has shaken Europe’s health systems, it has become clear that resources are scarce and need to be utilized to the best of their ability to ensure the viability and sustainability of the system.

This is a structure indicator. The index is a fraction where the numerator is the number of doctors, and the denominator is the number of beds in the clinic. The index is multiplied by a coefficient showing the average bed coverage of the clinic (mean completeness of the clinic). That is, for an average annual coverage of 80%, the ratio should be multiplied by 0.8 to increase the accuracy of the rating. The indicator may also be used to evaluate the staffing of the clinic, namely paramedical staff, and other professionals.

The index is used to evaluate staffing and find areas of under-staffing and possible over-staffing in the health system. It can be used for comparison between clinics of the same or different hospitals but also between hospitals in the same country or between different countries, especially between the EU Member States, where a target of performance is the convergence in policies for health systems and services.

#### 3.7.13. Economic Sustainability and Sustainable Development

Economic viability or sustainability refers to the strategic planning of the mental health system in such a way as to ensure endless financing and production of services, with the least possible risk of collapsing for the mental health services. Sustainable development additionally provides planning for the development and evolution of the system over time.

The Index is the answer YES/NO to the question of whether the mental health system has a viability study and whether the strategic planning of the system includes a separate chapter on strategic planning and sustainable development.

The performance target is the writing of a viability/sustainability study as well as a separate chapter on the sustainable development of the mental health system, in the strategic planning manual, which is also the benchmark. In addition to the health system, the indicator could be assessed at the level of the individual organizations included and constituting the system, but also at the level of hospitals and/or clinics.

#### 3.7.14. Average Beds Occupancy

The fullness of the beds in a clinic or hospital indicates that the available resources are being used to a sufficient extent but also indicate the need to expand the number of beds and, therefore, to increase staffing. This is a useful indicator that helps answer the question of how many doctors and nurses are needed for that particular clinic. The index is also particularly useful for comparisons between clinics of the same hospital and for comparisons between different hospitals.

This is a process indicator. The numerator is the total number of days of hospitalization per year of the clinic. This equals the number of beds, multiplied with patients’ average number of days in the hospital. The denominator is the theoretically maximum number of patient-days that this clinic could serve; that is, the number of beds in the clinic multiplies by 365 days of the year. A similar indicator is the average rotation interval, i.e., how many days a bed is left empty, which can be indirectly calculated from the average occupancy and number of beds.

The average occupancy rate of hospitals in European countries, which is 87%, can be used as a reference [39,40]. Occupancy below 80% can be a waste of resources, while occupancy 90% or above can lead the staff to occupational burnout syndrome as well as to increased hospital costs due to many on-call times. The purpose is to avoid having empty beds. Of course, this does not mean 100% occupancy, but certainly close to 90%, as there are several reasons a bed may be vacant (e.g., death, transfer to another clinic, cancellation of scheduled admission, or voluntary early departure from the clinic without the completion of the treatment plan).

#### 3.7.15. Average Inflow Rate (Average Number of Patients per Bed per Year)

The volume of work in a clinic is not only reflected by the average occupancy but also by the rate of rotation of patients in the beds. For example, two clinics with the same number of beds, where one hospital has twice the number of patients per year than the other.

This is an outcome indicator. The index is a fraction where the total number of patients treated in the clinic per year is the numerator, and the denominator is the number of beds in that clinic.

Based on published WHO data as a benchmark, bed needs can be calculated for an area depending on its population. In particular, in the WHO European Area, 60 beds and 453 patient admissions per year correspond for every 100,000 population. This corresponds to 7.6 patients per bed per year, i.e., 1.6 months or 48 days average hospital stay [28]. The index varies depending on the diagnosis and the severity of the cases being treated by a clinic. There is, of course, an indicative number of days of hospitalization for patients with a specific diagnosis, and this is described in the KEN/DRGs lists. By comparing clinical doctors with this indicator, each physician can try to converge to the average score of other clinicians of the same specialty. Specifically, for psychiatric clinics of the general hospitals, for Europe, the benchmark is 34.3 beds and 89.6 patients’ admissions per 100,000 population per year. Whereas, for the psychiatric hospitals, the corresponding benchmank is 12.3 beds and 160.5 patients’ admissions per 100,000 population per year [28].

#### 3.7.16. Average Bed Rotation Interval 

Making the most of the resources of a clinic and especially beds is not an easy task and requires a high degree of organization. Often some beds remain vacant and this reduces the utilization of the resources of the clinic. For example, when a clinic discharges patients on a daily basis, but admits patients only on specific days of the week, when the outpatient clinic and/or the Emergency Medicine Department of the hospital is working.

This is a process indicator. The indicator represents the days that the bed remains empty until the next patient arrives. The numerator is the number of empty-bed days (obviously may be greater than 365), and the denominator is the number of the beds of the clinic, multiplied by the 365 days of the year.

The goal of each clinic’s performance is to limit as much as possible the empty-bed days in the clinic, thereby reducing costs.

#### 3.7.17. Cost of Mental Health Services Compared to Health System Costs

The cost of health systems in the EU Member States is about 10% of each country’s GDP. A small percentage of this is attributed to mental health services.

The indicator shows the cost of mental health as a percentage of the country’s total health costs.

The indicator identifies unmet needs in mental health. It also points to areas where mental health is not yet a priority. Therefore, funding for the development of mental health services needs to be increased. There are no published benchmarks for this indicator. However, the indicator can be calculated indirectly, as there are published WHO data on governments’ per capita spending on mental health in each country. For the WHO European Region (54 countries) the per capita expenditure is US $21.7 per year, while the corresponding per capita expenditure globally is only US $2.5 [28]. According to the WHO report, in the European Union, the entire cost is covered by the state, with little or no cost to the patient for mental services [28].

#### 3.7.18. Existence of Strategic Plan and Action Plan

Strategic Plans and Action Plans are essential elements of health policy as they largely determine the quality of services in the present and the future. This indicator is also recommended by the WHO [7].

This is a process indicator. The indicator is qualitative and answers YES/NO to two key questions: (1) Is a written strategic plan available, and (2) is a written action plan available. These should be answered for the service as a whole and for each structure separately: (1) If there are timetables, (2) if there is written accountability at the end of each academic/clinical year, (3) if there is a fixed/global budget.

The performance goal is the answer YES to all queries. The budget should be separate for each structure, and it should be described as a percentage of the overall state budget for health. In addition, the expenditure on mental health should be specified as a percentage of the total expenditure of the health system (which includes both public and private mental health). It is not enough just to define budgets, but each individual budget must be accompanied by the sources of funding.

#### 3.7.19. Mental Health Research Index

The quality of mental health services can also be assessed by the number of ongoing research prjects, research protocols, doctoral theses and master theses, as a percentage of the total number of studies related to the health system. Published articles in scientific journals that use the peer review system should also be taken into account.

This is an outcome indicator. The indicator is the number of mental health studies, in relation to all health studies carried out in the country or health system or in the general hospital. The most important criterion is the overall impact of research on mental health, as shown by the overall impact factor of published mental health studies over a five-year period.

No benchmark has been published, but the aim is that mental health research should be at least quantitatively proportional to the percentage of funding for mental health services in relation to overall funding.

### 3.8. Safety Criterion or Safety Dimension. Indicators of Safety

#### 3.8.1. Number of Incidents of Verbal Violence

Verbal violence and verbal abuse of patients or staff are not permitted in mental health clinics. Patients who are admitted on a voluntary basis are usually informed in writing, and sign a written informed consent form, which commits them not to exhibit verbal or other violence against staff and patients, during their treatment in the inpatient ward and/or the outpatient clinic.

This is an outcome indicator. The indicator is a fraction where the numerator is the number of cases of verbal violence/abuse from patients to other patients or to clinical staff over the period of a year. The denominator is the total number of admissions during the same year.

There are no published performance targets for acceptable rates of verbal violence. The lower, the better.

#### 3.8.2. Number of Incidents of Physical Violence

Physical violence and/or verbal abuse to patients or staff is not permitted in mental health clinics, as in other clinics. Patients admitted to the clinic on a voluntary basis usually sign an informed consent document (informed consent) stating that they will avoid any form of violence during their hospitalization or treatment in the outpatient clinic.

This is an outcome indicator. The index is a fraction where the numerator is the number of cases of physical or verbal violence from patients to other patients or to staff during the year. The denominator is the number of patient admissions during the same time period.

There is no published acceptable rate of domestic violence cases per year. The objective is to continuously reduce the incidence of violence in terms of frequency, intensity, and duration.

#### 3.8.3. Number of Cases of Illegal Drug Use in the Clinic

Substance use disorder is a chronic, recurrent mental disorder. Co-morbidities are common, so patients may be referred—depending on the primary problem—to a general psychiatric clinic, or to more specialized clinics called detoxification and rehabilitation clinics, or other clinics for the treatment of dependence. It is not uncommon for a patient addicted to illicit euphoric substances to try to bring and use within the clinic the substance to which the patient is addicted (e.g., heroin). The frequency of these undesirable behaviors is an indicator of safety and quality of health care as they relate to the number, ability, training level, and experience of staff.

This is an outcome indicator. The indicator is a fraction where the numerator is the number of patients that showed positive urine tests for illicit substance use within the clinic during their hospitalization, and the denominator is the total number of patients admitted to the clinic during the same period.

No commonly accepted published data were found regarding an acceptable frequency of these adverse events. The less the better. The index can be used for the improvement of the clinic, year by year.

#### 3.8.4. Number of Cases of Alcohol Use in the Clinic

Alcohol use disorder is a chronic recurrent mental disorder. Patients often present with co-morbidities, so the clinic in which they will be hospitalized is selected on the basis of the pre-existing health problem, and may be the general hospital psychiatric clinic, the internal medicine clinic, or the alcohol detoxification and rehabilitation center. It is not uncommon for an alcohol-dependent patient to try and bring alcohol into the clinic. The frequency of such undesirable behaviors is an indicator of safety and quality of care and is related to the number of staff competence, suitability, training, and experience.

This is an outcome indicator. The index is a fraction where the numerator is the number of cases of alcohol use in the clinic, and the denominator is the total number of admissions in the clinic during the same period.

There is no published benchmark, but the index can be used with the aim of improving the quality of the clinic year after year.

#### 3.8.5. Personal Data Security

The General Data Protection Rule, as accepted and adopted by the European Parliament in 2016 [18], has brought about many changes in the data of patients with mental disorders, in the practice of psychiatry and other mental health professions, but also in the field of mental health in general. The patient’s personal data includes information such as name, age, place of residence, marital status, educational level, racial origin, political beliefs, religion, ideologies (political, philosophical), union, status health (diagnoses, treatments), erotic life and erotic preferences, criminal history (register of prosecutions and convictions). All of the above may be included in a mental health record, as they are often released as the patient answers open-ended and closed-ended questions during psychiatric interviewing and evaluation. Many of the above data are particularly important in forming the image of one’s personality. Therefore, these are regarded as sensitive personal data. Sensitive personal data are the health status (diagnoses, medications, hospitalizations), social welfare data (benefits, financial status), criminal history (prosecutions, convictions), ethnicity, nationality, citizenship, race, political choices, religious/philosophical beliefs, union, union, union (syndicate), erotic life (marital status, sexual preferences).

The General Data Protection Regulation regulates how health is collected, registered, organized, maintained/stored for a certain period, modified, exported, used, transmitted, disseminated, disposed of, linked to other databases, the combination of databases, databases connection, blocking, deletion, and destruction of personal data. It specifies in detail the conditions for the collection and processing of personal data, and in addition, for the sensitive personal data, it specifies that it may only be collected and processed after permission from the authorized Authority for the Protection of Personal Data. It also requires the consent of the subject whose data will be kept in the file.

This is a process indicator. The indicator determines whether the General Mental Health Regulation is followed in this mental health service and responds with YES/NO. Then, in free text format, detailed explanations can be given about areas of GDPR that do not apply to this structure/service and why.

The target of performance is the full implementation of GDPR in all Mental Health areas. In addition, another target is a yearly report that describes GDPR areas that are not being implemented, so that progress can be made until it is fully implemented.

#### 3.8.6. Existence of Institutionalized Internal and External Evaluation of Patients’ Human Rights

Patients’ rights are defined by international conventions, the United Nations (UN) and the European acquis. The assessment of the human rights situation of the mentally ill must be assessed in writing through statutory procedures at (1) at the local hospital/prison level, (2) at the national level, (3) at the international level. An important body of the Council of Europe, which assesses patients’ rights in compulsory treatment and detention structures, is the European Commission for the Prevention of Torture and Inhuman or Degrading Treatment or Punishment (CPT). The CPT pursues a policy of transparency by publishing on its website reports for each EU Member State it visits and evaluates [41].

This is a process indicator. The indicator is qualitative and receives the values YES/NO to questions whether there is an established written procedure for evaluating patients’ rights on a yearly basis (a) at a local intra-service level, (b) at national level (e.g., independent patient rights evaluation committee), (c) internationally (by an independent international organization). It should also be noted if these reports are published online so that they are transparent and accessible to patients and the general public.

In a WHO survey, only 28% of countries have regular evaluations on a yearly basis by a special commission or an independent body, which assesses whether legislation is in line with international human rights conventions (26). The performance target is to establish such a process, with timetables and transparency in the publication of evaluation findings and reports.

## 4. Discussion

Quality assessment in the provision of mental health services is a multidimensional process and difficult to implement. The difficulty is compounded by the lack of reliable biological markers in psychiatric evaluation, which makes it difficult to quantify any improvement after hospitalization, or in general after hospital or out-of-hospital pharmacotherapy and psychotherapy interventions [42]. The availability of biomarkers is going to increase in the near future as a great amount of research is already oriented in this direction [43].

The appropriateness of clinical structure and treatment is an important dimension of quality. Despite the progress in de-institutionalization in the mental health systems of most EU Member States, however, avoiding unnecessary admissions in the psychiatric clinic sometimes still remains a challenge. Additionally, a challenge is the continuity of rehabilitation in the community immediately after discharge from the hospital. For this to happen, appropriate outpatient structures are needed, which is not always possible, as many countries have been in a hurry to over-de-institutionalize, which has led to a deterioration in the prognosis and quality of life for a subgroup of chronic patients whose quality of life have decreased whilst morbidity has increased due to lack of appropriate community mental health services [44]. In this case, there is an unmet need for coordination and close co-operation between the general practitioners and community mental health services in order to improve appropriateness of referals [45]. The three indicators for qualitative assessment of appropriateness included in the review adequately quantify this dimension of services.

Accessibility to services is crucial for early treatment, and therefore for prognosis. Accessibility in mental health services is a core issue that affects all underprivileged people with mental health issues and is more evident in vulnerable subpopulations as the immigrants and refugees [46,47]. Accessibility also relates to cultural indicators such as stigma levels. Stigma has three dimensions: it is the set of negative mentalities, prejudice, and discrimination related to mental illness. The stigma comes from and concerns citizens in general and mental health professionals in particular and in particular the mentally ill and their relatives. The stigma discourages the patient from going to the doctor for early treatment, while later undermining the patient’s psychosocial recovery and social reintegration. High accessibility is related to less stigma and vice versa in the context of equity in healthcare, human rights, community involvement, continuity of care until patients’ recovery [48]. However, beyond stigma, accessibility also depends on the adequacy and availability of appropriate hospital and community mental health structures [44,49,50], as well as the absence of waiting lists for evaluations, hospitalizations, and outpatient therapies, as can be described.

In assessing the quality of mental health services, the degree of acceptability and overall opinion of service users is valuable and should be taken into account [51,52,53,54]. User evaluation is qualitative through interviews and complaint/suggestion boxes but also quantitative, through weighted questionnaires to which users can systematically respond, for example on the day of their dismissal from the clinic [55]. Factors that are playing an important role in forming patients’ opinion is respect and trust, feeling safety, information and explanation about clinical decisions, and family involvement in the treatment and rehabilitation process [56,57]. Particular attention should be paid to the evaluation by patients of their experience of hospitalization with regard to the degree of application of the General Data Protection Rule (GDPR).

The competence of mental health professionals is included in the evaluation and evaluated through the development of lifelong learning programs. There is increasing evidence and published guidance from the World Psychiatric Association (WPA) and European Psychiatric Association (EPA) regarding the importance of cultural competence for mental health professionals, in the current context of increased migration in the European Union as well as many other countries [58,59,60,61]. Additionally, of great importance is the development of competence for the treatment of sensitive populations, such as the sexual and gender minority populations [62]. In the field of Child and Adolescent Psychiatry, the availability of competent clinicians in the field of child sexual abuse is also considered to be of great importance [63].

The efficacy of therapeutic interventions is an important element of the quality of mental health services and is evaluated through indicators assessing the outcome of therapies and, more generally, the prognosis of patients with specific diagnoses and the structural characteristics of services.

Ensuring therapeutic continuity is an important element of quality and is evaluated both by the seamless referral, mobility, and accessibility of patients to the most appropriate health care services as well as by the smooth transition from inpatient care to outpatient services. Despite the debate between continuity of care versus specialization of teams, continuity of care has been related to better social functioning, especially when primary care and mental health care, addiction and social welfare services are oriented towards recovery [64,65,66].

Evaluating the effectiveness of mental health services is a multifaceted quality control process. It includes indicators of mainly economic interests that are important for the sustainability and sustainable development of structures, services, and the mental health system. Recruitment, funding, and resource utilization are important aspects that should be clearly included in the strategic planning and action plan of the organization.

Safety is an important element of quality and includes the safety of patients as well as health professionals. Violence in mainly verbal and rarely physical, and in danger are mainly male nurses who are working in the emergency psychiatry ward. Training in communication skills and de-escalating skills are important pillars of violence prevention in mental health services settings [56,67,68]. In addition to the security of individuals, the security of their personal data is of particular importance. Personal data relating to mental health services are by definition sensitive personal data. Therefore, their protection is strictly regulated by the EU General Data Protection Regulation.

The review includes the author’s most important quality indicators that cover all aspects of mental health service quality assessment. The current trend in measuring the quality of mental health has been taken into account, where research interest has now shifted from measuring structures and services to prognosis, outcome, and outcome measurement [69]. Nor has it escaped our attention that the over-focus on outcomes often overlooks populations with low access to mental health services, mainly due to their disadvantaged socio-economic status [70]. Efforts have been made so that the findings can be generalized in order to provide a tool for quality assessment and improvement in any health system.

However, it is a limitation of the study that that we did not search and, therefore, we did not include in this study, the body of published literature that did not have an English abstract. We considered only studies with an English abstract and we excluded those not referring to the developed countries, to the extent possible. The indicators presented in this study focus mainly on issues that are important in developed countries such as Cyprus, Greece and the rest of the EU Member States, rather than to poor underdeveloped or developing countries. Poor and underdeveloped countries may have other priorities, such as simply providing food and shelter/asylum for patients with reduced functionality due to mental disorders. Although even providing housing for the homeless with severe mental disorders is not sometimes a simple process, it is still a challenge even for the modern welfare state and the developed mental health services of wealthy European countries [71]. However, investing in mental health, regardless of the country’s GDP, has been described as having an excellent value-for-money relationship [72] and as a ‘best buy’ investment.

Another limitation of the study is the fact that it is not a systematic review but rather a narrative review, with a focus on clinically relevant indicators, that is, those that will ensure clinical quality. In particular, of the many indicators proposed in the literature, those with the greatest clinical relevance have been selected, which have as direct an impact as possible on improving the quality of mental health services enjoyed by the patient, and not on indicators that may have specialized economic, political or academic/research interest. Likewise, due to size limitations, no emphasis has been placed on preventive psychiatric and quality of life in healthy or non-clinical populations, despite the fact that such indicators are available in the literature since the importance of prevention is high.

In order to be used in practice, the above quality assessment indicators, for improvement of the quality of mental health services, they must be converted into electronic form, so that they can be automatically and systematically measured by appropriate e-health software. This will enable the indicators to be calculated automatically and their scores made available to the management team at any time. The existence of an integrated IT system for the collection of mental health data and indicators has been considered essential, and the establishment of a specialized independent European Mental Health Observatory within the EU has been proposed as an urgent need [73]. At Member State local level, in the context of administrative transparency in the use of resources now demanded by the public administration rules, it would be beneficial for index scores to be published each year on the Internet with open access to the public. This will not only ensure political transparency in the use of state resources, but it will also provide research data from authors working in the fields of health policy and planning. Future research directions in the may include the use of data envelopment analysis (DEA) methodology for the estimation of production frontiers and benchmarking of the mental health services [74] as well as the Grossman model of health demand of medical care [75], as already has been done in other fields of health studies. 

## 5. Conclusions

The long-term goal of this study is to provide an opportunity for Mental Health professionals to use research evidence, as should be expected, to underpin the public and mental health policy. A limited number of studies have so far partially tackled the problem of connecting the quality assessment issues with the performance of mental health systems [76,77,78]. This study provides a holistic approach to connect the quality aspects of a Mental Health System with its efficiency. Furthermore, the increasing availability of healthcare data will enhance the possibility of driving an important decision on how to build up a complex mental health system efficiently using Big data [79,80]. It is important to note that a set of indicators can be used simultaneously, and these can be used to frame the issues and to define the problems under consideration. Different criteria mean different problems. The indicators do not suggest a solution to the problem under consideration. Indicators collectively can help to clarify and quantify policy objectives and strategies for an optimized Mental Healthcare system.

Mental health is an important social parameter related to well-being, quality of life, human rights, but also to economics, creativity, productivity, sustainability, and sustainable development. Mental health is, therefore, socially good and the welfare state has a responsibility to maintain and improve it, through a system of mental health services of high quality, but also through interprofessional health education and preventive psychiatry programs. Improving the quality of mental health services requires assessing the existing quality and measuring and quantifying it so that comparisons can be made feasible over time at local, state, and transnational levels. Through the evaluation and measurement of quality with the proposed indicators, the areas of the mental health system that need to be emphasized and improved are revealed. Additionally, the optimization of available human resources and funding is optimized, and the mental health policies and planning of mental health services, based on scientific evidence, are encouraged.
